# The spectrum of Self-directed learning perceptions among faculty members and students of Bolan University of Medical and Health Sciences

**DOI:** 10.12669/pjms.38.7.6517

**Published:** 2022

**Authors:** Mukhtar Mehboob

**Affiliations:** 1 Mukhtar Mehboob, MBBS, FCPS, MRCS, FRCS (Glasgow), FACS (USA). Consultant Surgeon & Head of Surgery Department, Mohtarma Shaheed Benazir Bhutto General Hospital, Quetta, Pakistan

**Keywords:** Perceptions, SDL, Students, Faculty

## Abstract

**Objectives:**

To analyze the perception of students and faculty regarding self-directed learning (SDL).

**Methods:**

This mixed method study design (pragmatic) with both positivist and interpretive approaches was conducted on faculty members and students of Bolan Medical College Quetta from 1^st^ April 2021 to 31^st^ Aug 2021 on 246 medical and dental students and 12 faculty members. The research question was about the role of SDL in undergraduate medical students and faculty members. The inclusion criteria were any registered student and permanent faculty member, while exclusion criteria were any selected student and faculty member who not consented to participate in study. Data on self-directed learning instruments from medical students on 5 points Likert scale was collected by regular interval method and faculty members on an open narrative questionnaire by purposive sampling. The data were analyzed by the SPSS version 26, framework method of content analysis, and constant comparison technique.

**Results:**

The mean age of students was 21.45 + 2.01 years. There were 114 males and 132 females (ratio 1: 1.15). The maximum mean score (4.35) is 91.1% followed by (4.32) 84.0% of students’ percept that SDL constantly improves & excels in learning and successes and failures inspire SDL. The minimum mean score (3.32) 49.2% revealed that students felt difficulty in arranging and controlling their learning time. The faculty perception that SDL leads to professional identity (confidence & self-identity), improved technical skill (better expression & enhanced cognition), professionalism (focused learning, effective reflection, time management & self-satisfaction), collaboration, communicator, and leadership qualities.

**Conclusion:**

The students strongly believed that SDL will improve and excel but they need time management. Faculty members concluded that leadership, communication, collaboration, and professionalism are related to SDL.

## INTRODUCTION

The current trend has been changed from teacher-centered learning to student-centered learning (SPICES model).^1^ The Liaison Committee on Medical Education (LCME), an accrediting body for medical schools in the USA and Canada, directed medical schools to provide opportunities for students to participate in SDL.^2^ SDL is a methodology that provides lifelong learning in medical education.^3^ It initiates a learning motivation, planning & implementation, self-reflection, and interpersonal communication.^4^ In a study conducted by Khan A, et al. revealed that SDL improves the awareness among students, expand academic knowledge, polish practical skill and prepare them for professional skill.^5^ Learner equips themselves with time management, assignment preparation, examination preparation, note taking and effective use of information.

Historically, Knowles defined SDL as a process in which individuals take the initiative, with or without the help of others, in diagnosing their learning needs, formulating learning goals, identifying human and material resources for learning, choosing and implementing appropriate learning strategies, and evaluating learning outcomes.^6^ SDL is an extraordinary umbrella under which a considerable number of variables has been discussed, however, the feasibility of SDL in the different cultural environment has been the area of debate. Culture influences the learner’s ability to learn.^7^ How much student influence by the culture and inclusion of hybrid curriculum needs to be defined in detail? It was observed that students involved in the SDL curriculum had a significantly lower rating of knowledge as compared to the traditional curriculum.^8^ Franchi T et al. shared their experience about SDL that there is a distinct difference between students learned from SDL versus students learned from traditional method feeling unsure about whether they have achieved the desired learning outcomes.^9^ Strategic SDL training in the form of problem-based learning (PBL) and case-based discussion (CBD) is used to prepare future physicians for continuous professional development.

This study will identify the perception of faculty and students regarding the importance of SDL in medical education. The faculty perceptions make the institution able to go forward in pursuit of the development of curriculum, which provides in-built processes for SDL. Undergraduate students’ perception and readiness for SDL at a different level of study will enable researchers to motivate them for SDL linked with a modified curriculum, which would provide provisions to compel students to self-learning. The objectives were to analyze the perception of students and faculty regarding SDL. The study will further highlight the importance of new themes of SDL.

## METHODS

This mixed method design (pragmatic) with both positivist and interpretive approaches was conducted on faculty members and students of Bolan Medical College Quetta from 1^st^ April 2021 to 31^st^ August 2021. The quantitative sample size of students (n=246) was calculated by sampling error formula [n= Z^2^ P (1-P)/E^2^] confidence level of 95%, a margin of error of 5%, and a prevalence of 80%.^10^ Qualitative sample size of faculty members was twelve. The research question was about the role of SDL in undergraduate medical students and faculty members. The inclusion criteria were any registered student and permanent faculty member, while exclusion criteria were any selected student and faculty member who not consented to participate in study. Institutional review board approval was taken from Bolan University of Medical & Health Sciences Quetta (Ref. No. BUMHS/IRB/ 2021/12 Dated 22^nd^ Mar. 2021). The willingness or consent was signed by participants. Confidentiality was maintained.

The sampling technique for students was simple randomization by using a regular interval method and purposive sampling among faculty members till the same theme was recognized. The study participants were the faculty members and 1^st^, 3^rd^, 4^th^, final year MBBS students & 1^st^, 2^nd^, 3^rd^, and final year BDS students. The data was collected from the faculty members and undergraduate students on pre-designed proforma. The selected faculty members and students were briefed about study by the authors. Faculty members of dentistry and medicine were given the proforma to fill it in their convenient time and return back in next working day. Students were given the proforma in tutorial room and return back. We chose a questionnaire for our data collection to explore the individual’s experiences. The open-ended questionnaire for written answer was used for faculty members, while students’ response was recorded on five points Likert scale. A 20 items self-directed learning instrument (SDLI) described by Shen et al utilized; with answering on five points Likert scale was used (Crohn batch alpha was 0.91).^4^ The students were also provided with an opportunity to describe and explain their experiences. The questionnaire was not validated nor pilot study done but author was present at the time of student response to check the face and content validity of items and explain them where they feel difficulty. The same questionnaire was used for faculty narrative responses by using the ‘Framework method of content analysis. This method concludes both deductive (pre-defined codes) and inductive (emergent codes) approaches. Codes were refined and emergent themes were identified by a constant comparison approach. Themes were further reviewed by code co-occurrence and the relationship between the themes. We drew on the Standards for Reporting Qualitative Research (SRQR) and the Consolidated Criteria for Reporting Qualitative Research (COREQ) to guide our analysis and reporting of findings.

All the variables were analyzed by using the SPSS program (version 26). The SPSS was applied to analyze the social aspects of the study. Descriptive statistics were used for calculating frequencies, percentages, and means. The qualitative data obtained from faculty members were analyzed; subthemes and themes were identified.

## RESULTS

The mean age of students was 21.45 ± 2.01 years. There were 114 males and 132 females. The male to female ratio was 1: 1.15. There were 164 (66.7%) students from MBBS, 62 (37.80%) from pre-clinical group, while 102 (62.19%) from clinical group. There were 82 (33.3%) from BDS, 52(63.41%) from pre-clinical group, while 30 (36.59%) from clinical group. The overall pre-clinical group in study was 114 (46.34%) students, while clinical group 132 (53.66%).

The highest mean score (4.35) was recorded for item 3 “I strongly hope to constantly improve and excel in my learning” followed by a mean score (4.32) for item 4 “My successes and failures inspire me to continue learning.” Both items belonged to motivation. The lowest mean score (3.32) was recorded for item 11 “I am good at arranging and controlling my learning time”. The majority (91.1%) of the students were hopeful that SDL will improve and excel them, while only 49.2% were good at arranging and controlling their learning time (Table-I).

**Table I:** Perceptions of medical students regarding SDL.

S. No.	Items	Strongly Disagree (%)	Disagree (%)	Neutral (%)	Agree (%)	Strongly Agree (%)	Mean Score
***Learning Motivation***							
1	I know what I need to learn.	0.8	4.5	15	52	27.6	4.01
2	Regardless of the result or effectiveness of my learning, I still like learning.	2.0	14.6	14.6	41.9	26.8	3.76
3	I strongly hope to constantly improve and excel in my learning.	2.0	1.2	5.7	42.3	48.8	4.35
4	My successes and failures inspire me to continue learning.	1.2	3.3	8.5	35.8	51.2	4.32
5	I enjoy finding answers to questions.	1.6	1.6	11.8	47.6	37.4	4.17
6	I will not give up learning because I face some difficulties.	2.0	5.7	10.6	50.8	30.9	4.02
***Planning & Implementation***							
7	I can proactively establish my learning goals.	0.8	5.3	22.4	51.2	20.3	3.84
8	I know what learning strategies are appropriate for me in reaching my learning goals.	1.2	7.3	20.3	50.8	20.3	3.81
9	I set the priorities of my learning.	2.0	6.1	20.3	55.3	16.3	3.77
10	In the classroom or on my own, I can follow my plan of learning.	0.8	13.4	27.6	37.0	22.7	3.71
11	I am good at arranging and controlling my learning time.	5.3	19.9	25.6	35.8	13.4	3.32
12	I know how to find resources for my learning.	1.6	11.0	19.5	46.3	21.5	3.75
13	I can connect new knowledge with my personal experiences.	1.6	6.1	22.4	45.1	24.8	3.85
***Self-Monitoring***							
14	I understand the strengths and weaknesses of my learning.	4.0	5.3	18.3	55.7	20.3	3.9
15	I can monitor my learning progress.	0.4	10.2	19.1	56.5	13.8	3.73
16	I can evaluate my learning outcomes.	0.8	4.9	26.8	54.1	13.4	3.74
***Interpersonal Communication***							
17	My interaction with others helps me plan for further learning.	3.3	8.5	13.0	46.3	28.9	3.89
18	I would like to learn the language and culture of those whom I frequently interact with.	2.0	8.5	17.5	35.0	37.0	3.96
19	I can express messages effectively in oral presentations.	4.9	16.3	17.1	41.9	19.9	3.55
20	I can communicate messages effectively in writing.	3.3	6.1	16.7	47.6	26.4	3.87

The overall average motivation mean (4.10) score 82.18% (item 1 to 6), planning & implementation mean (3.72) score 66.41% (item 7 to 13), self-monitoring mean (3.79) score 69.23% (item 14 to 16) and interpersonnel communication mean (3.77) score 70.75% (item 17 to 20).

The faculty’s perception regarding the SDL was remarkable. The most interesting comments on confidence and self-identity were “SDL is very interesting & satisfying when I find the clues during my studies”, “SDL helps to improve my students’ confidence and skill level” and “SDL gives pleasure and confidence.” The interesting comments on better expression were “In SDL objectives are clear”, “SDL helps in recall and memorize learning effectively” and “SDL helped me to convey my views according to situation”. The comments on focused learning were “SDL requires colleagues for focused learning”, “I have changed my SDL strategy from books to article” and “SDL helps me to identify my knowledge gaps”.

They believed that SDL lead to professional identity (confidence & self-identity), improved technical skill (better expression & enhanced cognition), professionalism (focused learning, effective reflection, time management & self-satisfaction), collaboration, communication, and leadership qualities. Some faculty members emphasize the need for SDL among faculty members because they may act as role models in promoting medical students. (Table-II).

**Table II:** Deductive and inductive themes among faculty members.

S. No.	Items	Pre-defined themes (Deductive)	Codes Identified	Emergent subthemes	Themes (Inductive)
1	I know what I need to learn.	Learning motivation	Know learning material	Self-satisfaction	Confidence
			Don’t know the learning material	Learning leads to an innovative idea	
				Research updates	
2	Regardless of the result or effectiveness of my learning, I still like learning.	Learning motivation	Result & effectiveness	Results oriented	Self-identity
			Sense of satisfaction	Effectiveness	Better expression
				Practice	
3.	I strongly hope to constantly improve and excel in my learning.	Learning motivation	Improve & excel	Improve understanding	Improvement
			Improve but not excel	Practical implication lacking	Exaggerated improvement with practical implication
			Not improve & excel	SDL for clarity	
4	My successes and failures inspire me to continue learning.	Learning motivation	Success & Failure equal	Positive reinforcement	Stimulus leads to improve SDL
			More success less failure		
			Less success more failure	Negative reinforcement	
5	I enjoy finding answers to questions.	Learning motivation	Enjoy	Satisfaction Confidence	Enhance cognition
			Satisfied but not enjoyed	Satisfaction	
6	I will not give up learning because I face some difficulties.	Learning motivation	Not stop learning	Take alternative initiative	Enthusiasm
			Temporarily stop learning	Find alternate methods	Time line
			Stop learning	Time constraint	
7	I can proactively establish my learning goals.	Planning & Implementation	Definitive goal	Gain knowledge Clear confusion Students’ motivation	Focused learning
			No goal	Refreshment	Habituation
8	I know what learning strategies are appropriate for me in reaching my learning goals.	Planning & Implementation	Articles, books, internet, Slide share, image & MCQs	Target oriented Planned activity	Focused learning
9	I set the priorities of my learning.	Planning & Implementation	Problem oriented, role modeling, gain confidence and knowledge,	Focused learning	Effective reflection Timeline Confidence
10	In the classroom or on my own, I am able to follow my own plan of learning.	Planning & Implementation	Yes	Strategic planning	Leadership quality
			Yes, with difficulty		
11	I am good at arranging and controlling my learning time.	Planning & Implementation	Effective time utilization	Strict schedule for timeline	Time management
			Difficult to manage time	Feasibility	
			Time management till the goal achieved	Targeted approach	
12	I know how to find resources for my learning.	Planning & Implementation	Know	Access to learning resources	Collaboration enhance SDL
			Know with difficulty		
			Not find desired resources		
13	I can connect new knowledge with my own personal experiences.	Planning & Implementation	Enhance experience	Pleasure Confidence	Self-satisfaction
			Motivate for evidence base trial		
14	I understand the strengths and weakness of my learning.	Self-monitoring	Strength	Autonomy Dedication Confidence Hard work	Reflection in action
			Weakness	Fewer resources No competition No deadline Lack of focus No peer helps Timeline	
15	I can monitor my learning progress.	Self-monitoring	Can monitor	Discussion with colleagues and mentors	Reflection on action
			Can monitor with guidance		
16	I can evaluate on my own learning outcomes.	Self-monitoring	Can evaluate	Self- evaluation	Application of knowledge
			Not evaluate		
17	Whose interactions help you for further SDL e.g. peers, faculty members, friends, and family members?	Interpersonal communication	In order of frequently by faculty members, peers/friends, and family members	Motivation	External motivation
18	I would like to learn the language and culture of those whom I frequently interact with.	Interpersonal communication	In order of frequency not like, like, and like but not adopt	Motivation	External motivation
19	I can express messages effectively in oral presentations.	Interpersonal communication	All agreed	Expression	Collaborator
20	I can communicate messages effectively in writing.	Interpersonal communication	All agreed	Expression	Communicator

## DISCUSSION

The present study revealed that students know what learning strategies are appropriate for them in reaching their learning goals (mean score= 3.81).

In a study conducted by Yang C et al. there were 365 students from five medical colleges of China with male 152(41.64%) and female 213(58.36%).^7^ In present study there were 246 students from one medical and dental college with male 114 (46.53%) and 132(53.65%) females. There were also 12 faculty members. Imran M et al. conducted his qualitative study on four groups, which includes two pre-clinical and two clinical groups by conducting focus group discussion on 29 students of two to six years excluding foundation year.^11^ In our qualitative study on MBBS and BDS students the pre-clinical group had 114 (46.34%), while clinical group 132 (53.66%) students of 1-5 years of MBBS (excluding 2^nd^ year MBBS, their examination period) and one to four years of BDS. The purpose of including pre-clinical and clinical groups were to know the overall perception of SDL among students. This will help in assessing the current curriculum and determine the future needs by stakeholders.

Bhandari B et al. in their study conducted on medical students revealed that students scored high in SDL skill (mean score= 4.70) but they need improvement in time management skills (mean score= 3.79) and oral presentation skills (mean score= 3.74). Students also felt difficulty in finding resources for SDL (mean score= 3.86).^12^ In our study the students also scored high in SDL skills (mean score= 4.35). They face difficulty in time management (mean score= 3.32), oral presentation skill (mean score= 3.55) and finding resources for SDL (mean score= 3.75). There is a similarity between the findings of Bhandari B, et al and our study due to near same culture, educational environment and curriculum based medical education system. Akram A et al.; conceptualize the term non face to face (NF2F) student learning time, an ocean in medical education lead to improvement in self-student learning.^13^ Khalid M et al; in a survey revealed that SDL along with online learning had better academic performance as compared to conventional university student.^14^ In present study, students enjoy finding answer to their question (mean score= 4.17) and they connect new knowledge with their personal experiences (mean score= 3.85).

SDL established its understanding first as personal attributes (Moral, emotional and intellectual management), second as a process (Learners’ autonomy over instructions) and third as context (Environment where learning take place). The existing literature on SDL has established a good understanding of SDL as a process and personal attribute. The context, where learning occurs influences the autonomy of the learner. The enhancing opportunities include the availability of resources, a supportive environment, and leadership. The negative factors include a negative climate, restrictive policies, limited delegation of authorities that restrict initiatives, and constrained economic conditions.^15^ In our study students proactively establish their learning goals (mean score= 3.84). Conscientiousness, an informed acceptance of responsibility for one’s learning, and creativity in the form of artificial intelligence and visual reality techniques are keys to a student’s future orientation towards lifelong learning.^16^,^17^

Self-directed learning is defined here as learning habits demonstrated by an individual in taking charge of his learning activities with minimal assistance from others.^18^,^19^ In our study, some of the faculty considered SDL as a habit. Our faculty has a clear concept of how to monitor and evaluate SDL is very well understood. They are well aware of the strength and weaknesses of the SDL. Liu TH et al. revealed that a patient’s primary care activities act as a primary motivator for SDL activities.^20^ Our faculty members emphasized that practical implication provides a stimulus leading to improve SDL activities, enhanced cognition, and enthusiasm.

Ahmad N et al. in his study on multisource feedback (MSF) in young doctors contributes to increase the SDL.^21^ Faculty and peers also play a crucial role in guiding and promoting the SDL by offering feedback. Premkumar K et al. in his study revealed that faculty members indicated that society and parents have a lot of influence on students’ learning, while on other hand some students state that their parents motivate them to study and monitor them, while some said their parents are only concerned with their examination results and are not keen to know about their educational activities.^22^ In another similar study by Millanzi WC et al. it was revealed that facilitation in problem-based pedagogy helps in academic and professional achievements.^23^ In our study the students’ perception regarding interaction with others helps in SDL agreed by 75% of students, moreover, 72% want to adopt the culture and learn a language. The faculty believed that SDL leads to a good communicator and collaborator. A study by Hill M, et al. identified four themes of SDL, self-learning skill, collaboration, application, and meta-cognition.^10^ In our study new themes identified are communication, collaboration, leadership, professionalism, role modeling, and cognition.

### Strength of our study

The students of pre-clinical and clinical groups of both MBBS and BDS were included to know the overall perception of SDL of our institute. This will help in assessing the current curriculum and determine the future needs by stakeholders.

### Limitations of our study

The present study was based on a self-reported questionnaire that explored student abilities of SDL and, therefore, is not a direct measure of their SDL abilities. Psychometric analysis (aptitude and personality test) was not performed. The local institutional finding cannot be generalized. The new tools to determine SDL should be explored. Faculty must develop insight among students to connect their issues of learning with SDL and empower the students by providing facilities and comfort. Educational infrastructure should be strengthened to enhance SDL.

## CONCLUSION

The joint efforts by the facilitators and students themselves may be helpful to make students independent and lifelong learners. The students strongly believed that SDL will improve and excel but they need time management. Faculty members highlighted the themes like leadership, communicator, collaborator and professionalism related to SDL.
